# Gel-based proteomic map of *Arabidopsis thaliana* root plastids and mitochondria

**DOI:** 10.1186/s12870-020-02635-6

**Published:** 2020-09-04

**Authors:** Magda Grabsztunowicz, Anne Rokka, Irum Farooq, Eva-Mari Aro, Paula Mulo

**Affiliations:** 1 Molecular Plant Biology, University of Turku, 20520 Turku, Finland; 2 Turku Bioscience Centre, University of Turku and Åbo Akademi University, 20520 Turku, Finland

**Keywords:** 2-D gel electrophoresis, *Arabidopsis thaliana*, Mass spectrometry, Mitochondria, Proteomics, Root, Root plastid

## Abstract

**Background:**

Non-photosynthetic plastids of plants are known to be involved in a range of metabolic and biosynthetic reactions, even if they have been difficult to study due to their small size and lack of color. The morphology of root plastids is heterogeneous and also the plastid size, density and subcellular distribution varies depending on the cell type and developmental stage, and therefore the functional features have remained obscure. Although the root plastid proteome is likely to reveal specific functional features, *Arabidopsis thaliana* root plastid proteome has not been studied to date.

**Results:**

In the present study, we separated Arabidopsis root protein fraction enriched with plastids and mitochondria by 2D-PAGE and identified 84 plastid-targeted and 77 mitochondrion-targeted proteins using LC-MS/MS. The most prevalent root plastid protein categories represented amino acid biosynthesis, carbohydrate metabolism and lipid biosynthesis pathways, while the enzymes involved in starch and sucrose metabolism were not detected. Mitochondrion-targeted proteins were classified mainly into the energetics category.

**Conclusions:**

This is the first study presenting gel-based map of *Arabidopsis thaliana* root plastid and mitochondrial proteome. Our findings suggest that Arabidopsis root plastids have broad biosynthetic capacity, and that they do not play a major role in a long-term storage of carbohydrates. The proteomic map provides a tool for further studies to compare changes in the proteome, e.g. in response to environmental cues, and emphasizes the role of root plastids in nitrogen and sulfur metabolism as well as in amino acid and fatty acid biosynthesis. The results enable taking a first step towards an integrated view of root plastid/mitochondrial proteome and metabolic functions in *Arabidopsis thaliana* roots.

## B**ackground**

Plastids are plant cell organelles possessing diverse roles in energy metabolism, biosynthetic reactions and other metabolic activities. All plastid types are enclosed by a double envelope membrane and they contain several copies of a semiautonomous circular genome encoding circa 100 proteins, which are involved in photosynthesis, transcription and translation. However, a vast majority of plastid-localized proteins are encoded by a nuclear genome. These proteins are translated on cytosolic ribosomes and transported into the plastid using the information buried in the N-terminal transit peptide [1, 2]. The transit peptide selectively interacts with different translocon components hosted by a specific plastid, thereby discriminating between the plastids of different types and/or ages [1, 3]. The cell type and surrounding tissue together with environmental factors determine the direction of plastid development [4].

Plastids are divided in distinct groups depending on structure, (pigment) composition and functional properties. The best characterized plastid type is the chloroplast, which are abundant in green tissues. Chloroplasts are the sites of photosynthesis and contain an elaborate internal membrane system called the thylakoids, in which the large pigment-protein complexes involved in photosynthetic electron transfer reactions are embedded. Flowers and fruits contain chromoplasts, which are rich in yellow and orange carotenoids, while roots (and other non-photosynthetic tissues) contain leucoplasts, which can be further classified as elaioplasts storing lipids, proteinoplasts containing crystalline protein bodies, and amyloplasts storing starch [5]. Chloroplasts are able to capture light energy and convert it into chemical form as ATP and reducing equivalents of NADPH, while the non-photosynthetic plastids are dependent on the import of external sugar phosphates and ATP to the plastid.

The non-photosynthetic plastids are involved in a range of metabolic and biosynthetic reactions, such as biosynthesis of starch [6], carotenoids [7] and lipids [8] as well as assimilation of nitrogen [9]. Although the proteome of a certain plastid type is likely to reflect and reveal specific functional features, there are limited data available on the proteomes of non-photosynthetic plastids. Thus far, the proteomes of potato tuber amyloplasts [10], wheat endosperm amyloplasts [11, 12], chromoplasts from bell pepper and sweet orange fruits [13, 14], non-differentiated plastids from tobacco cell culture [15], rice etioplasts [16], and *Medicago truncatula* root plastids [17, 18] have been studied, while there is no information available on the root plastid proteome of *Arabidopsis thaliana*.

In the present study, we have taken the same strategy used in most studies focusing on non-photosynthetic plastid proteomes: enrichment of root plastids, separation of the proteins using 2D-gel electrophoresis followed by in-gel digestion and identification of the protein spots using LC-MS/MS [e.g. 10, 11, 17]. The identified proteins reveal a complex regulatory and metabolic network present in *Arabidopsis thaliana* root plastids and mitochodria, and enable a first step towards an integrated view of root plastid/mitochondrial proteome and metabolic functions of roots.

## Results

**Fig. 1:**
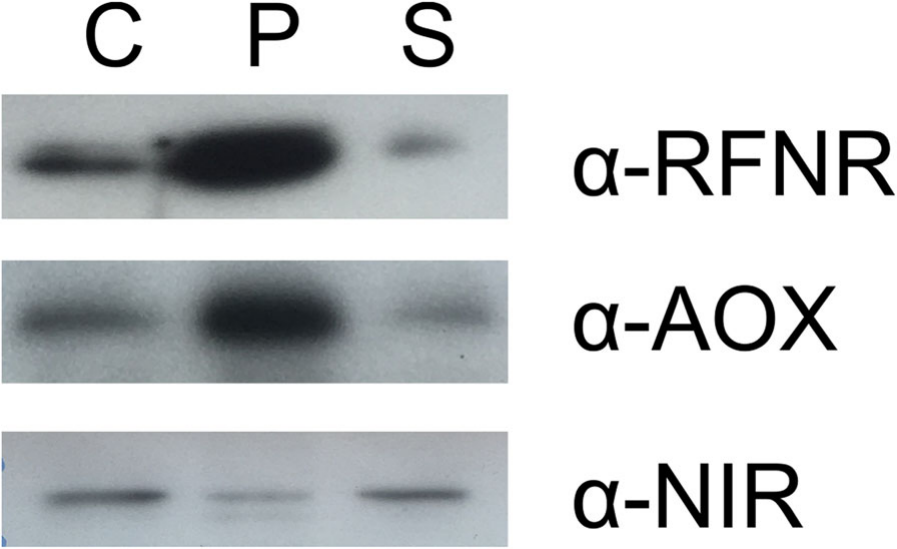
Analysis of the root protein sample. 20 μg of crude root protein extract (C), the pellet representing root plastids (P) and supernatant representing cytosol (S) were separated by SDS-PAGE, transferred to a PVDF membrane and immunolabelled with root-type FNR (RFNR; root plastid marker), alternative oxidase (AOX1/2; mitochondrial marker) and nitrate reductase (NR; cytosolic marker) antibodies

Due to the small size (diameter of root plastids ranging from 1.8 to 3.0 μm as compared to 5–10 μm in chloroplasts) and lack of color, root plastids have been difficult to study, and therefore they are still poorly characterized [19]. Nevertheless, it has been shown that the morphology of root plastids is very heterogeneous and the plastid shape (often described as amoeboid), size, density and subcellular distribution varies depending on the cell type and the stage of development [19]. Additionally, root plastids are abundant in stromules, the stroma filled tubules which can interconnect different plastids [20]. As the size of mitochondria (0.1–0.5 μm × 1–2 μm) falls in the same range as the root plastids, it is not surprising that the sample representing enriched root plastids also contained mitochondria, with a minor contamination by cytosolic proteins (Fig. 1; Additional file 1: Figure S1). These contaminants are frequently detected also in other studies using the same approach [10, 11, 17]. The preparation was further used to study the leucoplastidic and mitochondrial proteome of Arabidopsis roots.

**Fig. 2:**
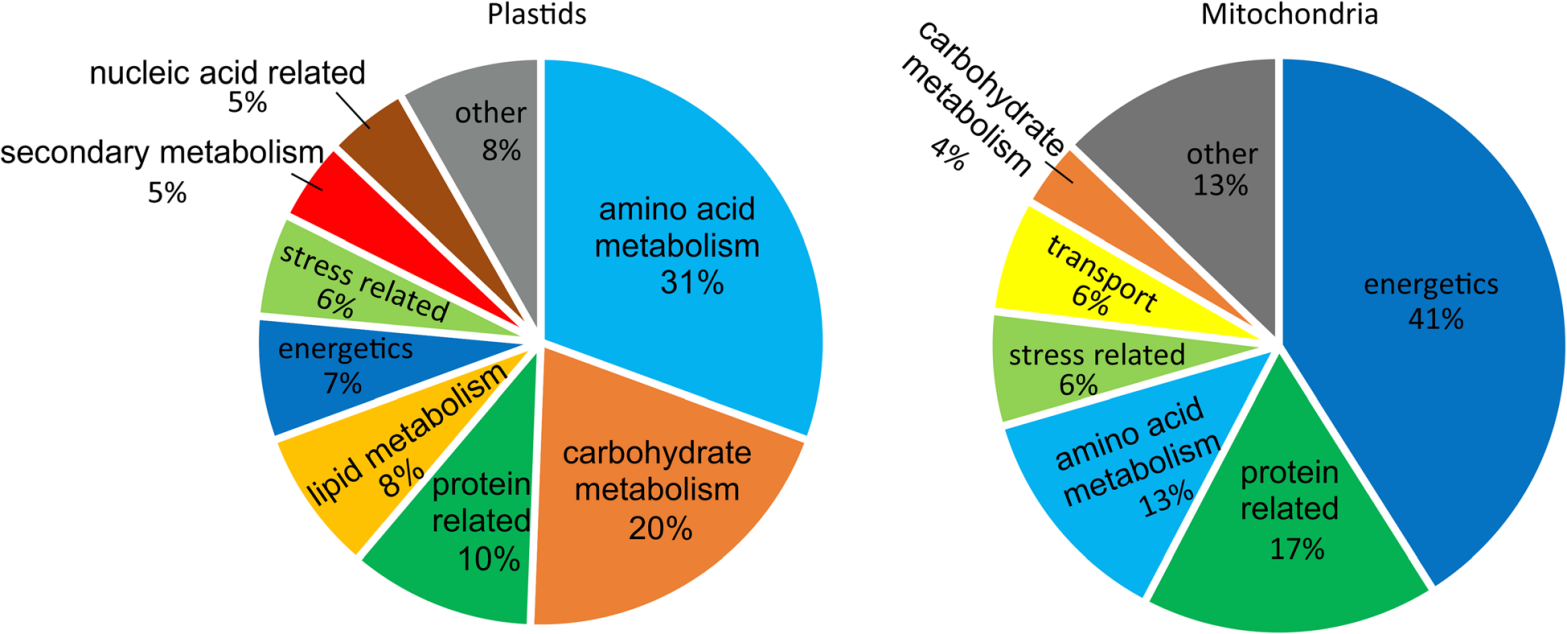
Classification of Arabidopsis root proteins from a fraction enriched with plastids and mitochondria. The classification is based on UniProt database and references indicated in text. Numbers in percentages (%) correspond to the numbers of gene ontology (GO) terms assigned for particular GO category

Separation of organellar root proteins by isoelectric focusing using the pH range from 3 to 11 followed by SDS-PAGE and Sypro Ruby staining revealed 139 distinct protein spots that could be identified in at least three independent biological replicates (Additional file 2: Figure S2). We selected the spots for LC-MS/MS analysis based on the following criteria: (i) the spots were clearly visible in three biological replicates, (ii) the spots were separated from neighboring spots, and (iii) the spots were not too big and intensive, because such spots usually contain a high number of different proteins. Low molecular weight protein spots were excluded, as they usually do not provide enough peptides for protein identification. Moreover, some spots showing the same molecular weight but different pI were selected (e.g. 1–4, 6 and 7–9 in Additional file 2: Figure S2 A), because we wanted to see if these spots represented different isoforms of the same protein. The proteins identified with high confidence and represented by at least 3 peptides, expected molecular weight and isoelectric point (sequence based predictions) are presented in Additional file 3: Table S1. The plastid-targeted proteins were classified into nine, and the mitochondrion-targeted proteins into seven functional categories (Fig. 2) based on localizations and functions annotated in UniProt and/or according to recent literature (e.g. [12] and other references presented in text). In plastids, the most prevalent categories represented amino acid biosynthesis (31%) and carbohydrate metabolism (20%), while in mitochondria the proteins involved in energetics (41%) formed the largest category (Fig. 2). It should be noted, however, that the proteins not included in these categories may still have an (unknown) role in these pathways, or have other important roles in root metabolism.

### Amino acid biosynthesis pathways in root plastids

**Fig. 3:**
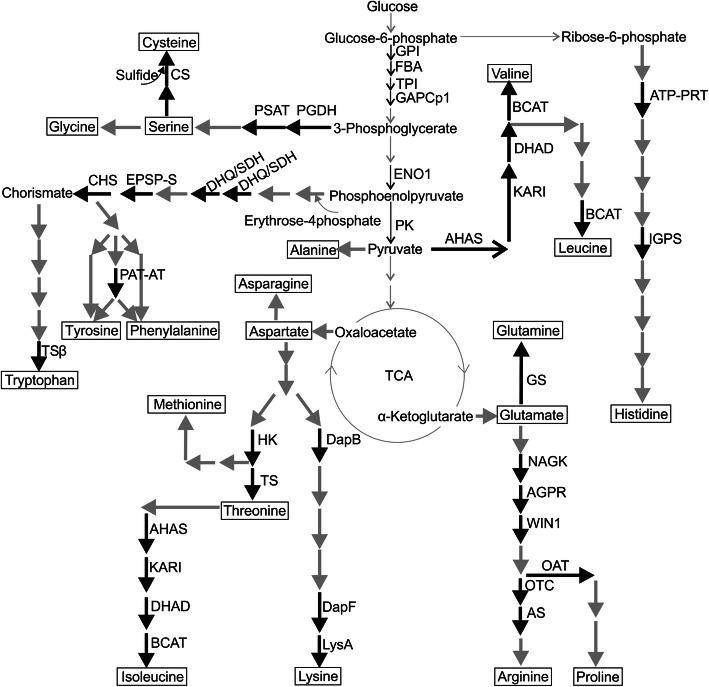
Simplified scheme of the amino acid biosynthesis pathways and glycolysis in plants. Black arrows represents the reactions catalysed by the enzymes identified in plastid enriched fraction, grey arrows indicate reactions catalysed by the enzymes which were not detected in the current work. AHAS, acetolactate synthase small subunit; 1AGPR, probable N-acetyl-gamma-glutamyl-phosphate reductase; AS, argininosuccinate synthase; ATP-PRT, ATP phosphoribosyltransferase; 1BACAT, branched-chain-amino-acid aminotransferase; 3CHS, chorismate synthase; CS, cysteine synthase; DapB, 4-hydroxy-tetrahydrodipicolinate reductase; 1DapF, diaminopimelate epimerase; DHAD, dihydroxy-acid dehydratase; DHQ-SDH, bifunctional 3-dehydroquinate dehydratase/shikimate dehydrogenase; EPSP-S, 3-phosphoshikimate 1-carboxyvinyltransferase; FBA, fructose-bisphosphatase aldolase; GAPCp1, plastid localized glyceraldehyde-3-phosphate dehydrogenase; GPI, glucose-6-phosphate isomerase 1; HK, homoserine kinase; IGPS, imidazole glycerol phosphate synthase; KARI, ketol-acid reductoisomerase; LysA, diaminopimelate decarboxylase; NAGK, acetylglutamate kinase; OAT, ornithine aminotransferase; OTC, ornithine carbamoyltransferase; PAT-AT, bifunctional aspartate aminotransferase and glutamate/aspartate-prephenate aminotransferase; PGDH1, D-3-phosphoglycerate dehydrogenase; PSAT, phosphoserine aminotransferase; TCA, tricarboxylic acid cycle; TPI, triose phosphate isomerase; TS, threonine synthase; TSβ, tryptophan synthase beta chain; WIN1, acetylornithine aminotransferase

Enzymes involved in the biosynthesis of amino acids were well represented among the Arabidopsis root plastid proteins (Figs. 2, 3, Table 1). First of all, three enzymes involved in diaminopimelic acid (DAP)- dependent pathway of L-lysine synthesis were identified: 4-hydroxy-tetrahydrodipicolinate reductase (DapB, At2g44040, spot 86) catalyzing formation of tetrahydrodipicolinate from dihydrodipicolinate, diaminopimelate epimerase (DapF, At3g53580, spot 86) forming meso-diaminoheptanedioate from LL-2,6-diaminoheptanedioate, and diaminopimelate decarboxylase 2 (LysA, At5g11880, spot 56), the enzyme catalyzing the last step of the pathway where the L-lysine is synthetized from meso-diaminoheptanedioate [21]. Moreover, homoserine kinase (HK, At2g17265) and threonine synthase 1 (TS, At4g29840), the enzymes involved in L-threonine synthesis [22], were found in the plastid enriched fractions. HK (present in spots 83 and 85) forms homoserine phosphate from homoserine and inorganic phosphate, while the TS (detected in spots 48, 49 and 50) catalyzes the pyridoxal 5′-phosphate dependent conversion of L-homoserine phosphate into L-threonine and inorganic phosphate [23].

**Table 1:** Annotated root plastid proteins identified by mass spectrometric analysis

Function	Spot number	Accession (UP), Gene locus (TAIR)
**AMINO ACID METABOLISM**		
*Synthesis*		
3-phosphoshikimate 1-carboxyvinyltransferase	51, 52	P05466, At2g45300
4-hydroxy-tetrahydrodipicolinate reductase 1	86	O80574, At2g44040
Acetolactate synthase small subunit 1	58	Q9FFF4, At5g16290
Acetylglutamate kinase	92	Q9SCL7, At3g57560
Acetylornithine aminotransferase	62, 63	Q9M8M7, At1g80600
Argininosuccinate synthase	51, 52	Q9SZX3, At4g24830
ATP phosphoribosyltransferase 1	62	Q9S762, At1g58080
Bifunctional 3-dehydroquinate dehydratase/shikimate dehydrogenase	30	Q9SQT8, At3g06350
Bifunctional aspartate aminotransferase and glutamate/aspartate-prephenate aminotransferase	64, 66	Q9SIE1, At2g22250
Branched-chain-amino-acid aminotransferase 3	64, 66	Q9M401, At3g49680
Chorismate synthase	71, 73	P57720, At1g48850
Cysteine synthase	82, 83, 102	P47999, At2g43750
D-3-phosphoglycerate dehydrogenase 1	18, 22–24, 30, 45–47, 54, 55	O49485, At4g34200
Diaminopimelate decarboxylase 2	56	Q94A94, At5g11880
Diaminopimelate epimerase	86	Q9LFG2, At3g53580
Dihydroxy-acid dehydratase	30, 43, 45	Q9LIR4, At3g23940
Glutamine synthetase	55	Q43127, At5g35630
Homoserine kinase	83, 85	Q8L7R2, At2g17265
Imidazole glycerol phosphate synthase hisHF	43–46, 56	Q9SZ30, At4g26900
Ketol-acid reductoisomerase	36, 37	Q05758, At3g58610
Ornithine carbamoyltransferase	85	O50039, At1g75330
Phosphoserine aminotransferase 1	64, 66	Q96255, At4g35630
Phosphoserine aminotransferase 2	66	Q9SHP0, At2g17630
Probable N-acetyl-gamma-glutamyl-phosphate reductase	70, 71	Q93Z70, At2g19940
Threonine synthase 1	48–50	Q9S7B5, At4g29840
Tryptophan synthase beta chain 1	65	P14671, At5g54810
*Regulation*		
ACT domain-containing protein	88	Q9LZ23, AT5G04740
**CARBOHYDRATE METABOLISM**		
*Glycolysis*		
Dihydrolipoyllysine-residue acetyltransferase component 4 of pyruvate dehydrogenase complex	46, 47, 63	Q9SQI8, At3g25860
Dihydrolipoyllysine-residue acetyltransferase component 5 of pyruvate dehydrogenase complex	47, 48, 50	Q9C8P0, At1g34430
Enolase 1	51, 52	Q9C9C4, At1g74030
Fructose-bisphosphate aldolase 3	76, 83, 85, 87	Q9ZU52, At2g01140
Glucose-6-phosphate isomerase 1	25, 30	Q8H103, At4g24620
Glyceraldehyde-3-phosphate dehydrogenase	71, 75, 82, 85	Q9SAJ6, At1g79530
Isocitrate dehydrogenase [NADP]	65	Q8LPJ5, At5g14590
Phosphoglucomutase	22	Q9SCY0, At5g51820
Plastidial pyruvate kinase 1	22, 23, 79	Q9LIK0, At3g22960
Plastidial pyruvate kinase 2	47	Q9FLW9, At5g52920
Triosephosphate isomerase	101	Q9SKP6, At2g21170
*Pentose Phosphate Pathway*		
Transketolase-1	13	Q8RWV0, At3g60750
Transketolase-2	20, 54	F4IW47, At2g45290
*Starch Synthesis*		
Glucose-1-phosphate adenylyltransferase small subunit	52	P55228, At5g48300
*Others*		
Malate dehydrogenase	82, 83	Q9SN86, At3g47520
Ribulose bisphosphate carboxylase large chain	27, 49	O03042, AtCg00490
**PROTEIN RELATED**		
*Degradation*		
ATP-dependent zinc metalloprotease FTSH 2	14	O80860, At2g30950
Chaperone protein ClpC2	8, 9	Q9SXJ7, At3g48870
Leucine aminopeptidase 2	47	Q944P7, At4g30920
Leucine aminopeptidase 3	47	Q8RX72, At4g30910
*Folding*		
Chaperonin 60 subunit alpha 1	22, 24, 25, 27, 28, 30, 49	P21238, At2g28000
Chaperonin 60 subunit beta 2	22, 23, 28, 89	Q9LJE4, At3g13470
Chaperonin 60 subunit beta 3	28, 30	C0Z361, At5g56500
Peptidyl-prolyl cis-trans isomerase CYP37	79	P82869, At3g15520
*Synthesis*		
Glutamyl-tRNA (Gln) amidotransferase subunit A	46, 47	Q9LI77, At3g25660
**LIPID METABOLISM**		
3-oxoacyl-[acyl-carrier-protein] reductase	97	P33207, At1g24360
3-oxoacyl-[acyl-carrier-protein] synthase I	59, 85	P52410, At5g46290
Acetyl-coenzyme A carboxylase carboxyl transferase subunit beta	47, 59	P56765, AtCg00500
Biotin carboxyl carrier protein of acetyl-CoA carboxylase 1	86	Q42533, At5g16390
Biotin carboxylase	47	O04983, At5g35360
Enoyl-[acyl-carrier-protein] reductase [NADH]	82, 83	Q9SLA8, At2g05990
Pyruvate dehydrogenase E1 component subunit beta-3	79	O64688, At2g34590
**ENERGETICS**		
*ATP synthesis*		
ATP synthase subunit alpha	22, 30, 49, 86	P56757, AtCg00120
ATP synthase subunit beta	43–45, 49	P19366, AtCg00480
*Electron Transfer*		
Adenylate kinase 2	102	Q9FIJ7, At5g47840
Ferredoxin-NADP reductase, root isozyme 1	77	Q9M0V6, At4g05390
Ferredoxin-NADP reductase, root isozyme 2	78	Q9S9P8, At1g30510
**STRESS RELATED**		
*Abiotic Stress Response*		
Heat shock 70 kDa protein 6	12, 13, 24	Q9STW6, At4g24280
Heat shock 70 kDa protein 7	12, 13	Q9LTX9, At5g49910
Probable plastid-lipid-associated protein 1	88	O81439, At4g04020
*Redox Regulation*		
Glutathione reductase	49, 50	P42770, At3g54660
Glutamate-cysteine ligase	51, 52	P46309, At4g23100
**SECONDARY METABOLISM**		
3-isopropylmalate dehydratase large subunit	48–50	Q94AR8, At4g13430
4-hydroxy-3-methylbut-2-enyl diphosphate reductase	52	Q94B35, At4g34350
Isopentenyl-diphosphate delta-isomerase II	89	Q42553, At3g02780
Protein strictosidine synthase-like 12	82	P94111, At1g74020
**NUCLEIC ACID RELATED**		
Adenylosuccinate synthetase	56–58	Q96529, At3g57610
Carbamoyl-phosphate synthase small chain	52	Q9LVW7, At3g27740
Dihydropyrimidine dehydrogenase	56, 64, 65	Q9LVI9, At3g17810
Soluble inorganic pyrophosphatase 6	88	Q9LXC9, At5g09650
**NITROGEN METABOLISM**		
Ferredoxin-nitrite reductase	45, 51, 52	Q39161, At2g15620
**C1 METABOLISM**		
Bifunctional protein FolD 4	71, 75	O65271, At4g00620
iron sulfur cluster biosynthesis		
SufE-like protein 1	79	Q84W65, At4g26500
**TETRAPYRROLE SYNTHESIS**		
Delta-aminolevulinic acid dehydratase 1	54, 55	Q9SFH9, At1g69740
**SULFUR ASSIMILATION**		
ATP sulfurylase 1	56, 57	Q9LIK9, At3g22890
**PLASTID ORGANIZATION**		
3-isopropylmalate dehydratase small subunit 3	90, 102, 109	Q9ZW85, At2g43090

Also enzymes involved in the biosynthesis of serine, cysteine and histidine were identified (Table 1, Fig. 3). D-3-phosphoglycerate dehydrogenase (PGDH1, At4g34200), the first enzyme in the plastidial phosphorylated pathway of L-serine biosynthesis, was identified in multiple spots (18, 22–24, 30, 45–47, 54 and 55). It converts 3-phosphoglycerate to 3-phosphohydroxypyruvate, which is further transformed to O-phosphoserine by phosphoserine aminotransferase (PSAT). The two PSAT isoforms encoded by the Arabidopsis genomes were both detected in our experiment, PSAT 1 (At4g35630) in spots 64 and 66 and PSAT 2 (At2g17630) in spot 66. Additionally, plastidial cysteine synthase (CS, At2g43750), catalyzing conversion of O-acetylserine and sulfide to L-cysteine and acetate, was found in spots 82, 83 and 102. ATP- phosphoribosyltransferase (ATP-PRT, At1g58080, spot 62) and imidazole glycerol phosphate synthase (IGPS, At4g26900, spots 43–46 and 56) are involved in L-histidine biosynthesis. ATP-PRT catalyzes the condensation of ATP and 5′-phosphoribosyl-1-pyrophospathe (PRRPP) to N′-5’phosphoribosyl-ATP (PRATP), while IGPS is involved in conversion of aminoketose PRFAR (phosphoribosyl formimo-5-aminoimidazole-4-carboxyamide ribonucleotide) to imidazole glycerol phosphate [24].

The L-arginine biosynthesis pathway was well represented, as five enzymes of the pathway were identified from our root plastid sample (Table 1, Fig. 3). Acetylglutamate kinase (NAGK, At3g57560), catalyzing phosphorylation of N-acetylglutamate was identified in spot 92. N-acetylglutamate phosphate is then converted to N-acetylglutamate semialdehyde by N-acetylglutamyl-phosphate reductase (AGPR, At2g19940, spots 70 and 71). In the next step, acetylornithine aminotransferase (WIN1, At1g80600), detected in spots 62 and 63, transfers an amino group from a second glutamate to form N^2^-acetylornithine. Subsequently, the produced ornithine is further used by ornithine transcarbamomylase (OTC, At1g75330, spot 85) to form citrulline. Argininosuccinate synthase (AS, At4g24830) detected in spot 51 and 52 converts L-citrulline to argininosuccinate that is the direct substrate for synthesis of L-arginine [25].

The branched-chain amino acids (BCAAs), namely L-isoleucine, L-valine and L-leucine, belong to the nine of 20 amino acids that cannot be synthetized de novo by humans nor animals. The BCAAs synthesis is known to take a place inside of plastids [26], and indeed all four enzymes catalyzing subsequent steps in conversion of L-threonine or pyruvate to different BCAAs were identified in our sample enriched with root plastids (Table 1, Fig. 3). Acetolactate synthase (also known as acetohydroxyacid synthase, AHAS, spot 58) is the first enzyme in the BCAA synthesis pathway that catalyzes the condensation of two molecules of pyruvate to 2-acetolactate or condensation of pyruvate and α-ketobutyrate to 2-aceto-2-hydroxybutyrate. In plants, AHAS works as a heterodimer formed by a catalytic and a regulatory subunit [27, 28]. In Arabidopsis, two genes for the regulatory subunits have been identified, and one of them (At5g16290) was detected in spot 58. Ketolacid reductoisomerase (KARI, At3g58610, present in spots 36 and 37) isomerizes and then reduces two acetohydroxyacids to produce dihydroxyacids. This reaction is the second step in the parallel pathway towards L-valine and L-isoleucine biosynthesis [22, 29]. Dihydroxyacid dehydratase (DHAD, At3g23940), performing the third step in synthesis of L-isoleucine from 2-oxobutanoate or synthesis of L-valine from two molecules of pyruvate [30], was identified in spots 30, 43 and 45. Finally, one of the three plastid localized isoforms of branched-chain-amino-acid aminotransferase (BCAT At3g49680) [31] was detected in spots 64 and 66. BCATs catalyze the last step of all the three BCAA biosynthesis pathways, converting the respective α-keto acids to amino acids [32].

The Shikimate pathway is a multistep metabolic route that converts the primary metabolites phosphoenolpyruvate (PEP) and erythrose-4-P to chorismate. Chorismate is further used as a precursor for synthesis of the three aromatic amino acids (L-phenylalanine, L-tyrosine, and L-tryptophan) as well as other aromatic compounds (like pigments and hormones) required for plant growth, development and defense [33]. In our studies, we have identified three enzymes involved in the shikimate pathway, the bifunctional 3-dehydroquinate dehydratase/shikimate dehydrogenase (DHQ/SDH; At3g06350, in spot 30), 5-enolpyruvylshikimate-3-phosphate (EPSP) synthase (also referred as 3-phosphoshikimate 1-carboxyvinyltransferase; At2g45300, spots 51, 52) and chorismate synthase (CHS, At1g48850, spots 71, 73) (Table 1, Fig. 3). The first enzyme catalyzes two subsequent reactions in the pathway leading to conformation of 3-dehydroquinate into shikimate [34]. EPSP synthase transfers an enolpyruvoyl moiety from PEP to shikimate 3-phosphate via a chemically unusual reaction involving C-O bond cleavage of PEP rather than P-O bond cleavage as in most PEP-using enzymes [35, 36]. The product of this reaction, EPSP, is further transformed to chorismate via 1,4-trans elimination of the phosphate and hydrogen by chorismate synthase.

In plants, several different enzymes compete for chorismate in post-chorismate metabolic pathways. In one of these metabolic branches, chorismate is converted into L-tryptophan. The last two steps of this pathway are catalyzed by tryptophan synthase α subunit and β subunits, respectively. Tryptophan synthase subunit β (TSβ, At5g54810) was detected in spot 65, corroborating the earlier results suggesting that in addition to green parts of the plant, L-tryptophan is also synthesized in Arabidopis roots [37, 38]. Also, the post-chorismate metabolic branch that leads to synthesis of L-phenylalanine and L-tyrosine was represented in root plastid samples, as the bifunctional aspartate aminotransferase and glutamate/aspartate-prephenate aminotransferase (PAT-AT; At2g22250) [39] was identified in spots 64 and 66 (Table 1, Fig. 3).

### Carbohydrate metabolism and assimilatory pathways

Twenty percent of the identified plastidial proteins represented carbohydrate metabolism, including several enzymes involved in glycolysis. Glucose-6-phosphate isomerase 1 (GPI, At4g24620), found in spots 25 and 30, is a dimeric enzyme that catalyzes reversible isomerization and interconversion of glucose-6-phosphate and fructose-6-phosphate. Fructose-bisphosphatase aldolase (FBA 3; At2g01140 detected in spots 76, 83, 85, 87), known to be involved in condensation reaction of fructose-1,6-biphosphate and sedoheptulose-1,7-biphosphate in the Calvin cycle [40], also catalyzes a reversible reaction in the glycolytic pathway splitting fructose 1,6-bisphosphate into dihydroxyacetone phosphate (DHAP) and glyceraldehyde 3-phosphate (G3P) [41]. Triose phosphate isomerase (TPI, At2g21170, spot 101) transforms DHAP into G3P, which is further converted to 1,3-bisphosphoglycerate by the glyceraldehyde-3-phosphate dehydrogenase enzyme (GAPDH), with the simultaneous reduction of NAD^+^ to NADH. GAPCp1 (At1g79530, spots 71, 75, 82, 85) is one of the two plastid-localized glycolytic isoforms of GAPDH (in addition to the two cytosolic isoforms) present in Arabidopsis [42, 43]. Regeneration of NAD^+^ is catalyzed by the NAD-dependent malate dehydrogenase (MDH; At3g47520, spots 82 and 83), which is responsible for the interconversion of oxaloacetate and malate [44]. Enolase 1 (ENO1, At1g74030), which catalyzes the conversion of 2-phosphoglycerate (2-PG) to PEP, was detected in spots 51 and 52. Plastidial pyruvate kinase 2 (PK, At5g52920), detected in spot 47, functions in the last steps of glycolysis. It catalyzes the transfer of a phosphate group from PEP to adenosine diphosphate (ADP), yielding one molecule of pyruvate and one molecule of ATP. Next, pyruvate dehydrogenase complex, composed of several copies of at least four enzymes, performs transformation of pyruvate into acetyl-CoA (pyruvate decarboxylation). The dihydrolipoyl-lysine residue acetyltransferase components 4 (At3g25860) and 5 (At1g34430) of pyruvate dehydrogenase complex were detected in spots 46, 47, 63, and 47, 48 and 50, respectively, while E1, the beta-3 subunit of pyruvate dehydrogenase (At2g34590) was detected in spot 79 (Table 1). Acetyl-CoA, in turn, is further used in various processes, such as fatty acid biosynthesis (see below).

In non-photosynthetic plastids (as well as in chloroplasts during the dark period), the oxidative pentose phosphate pathway generates reducing power in the form of NADPH to be used in numerous metabolic pathways, including nitrogen and sulfur assimilation. NADPH is oxidized by the root-type ferredoxin-NADP^+^ oxidoreductase enzymes (RFNR1, At4g05390 in spot 77 and RFNR2, At1g30510 in spot 78), which subsequently reduce root-type ferredoxin proteins [45]. Ferredoxin-nitrite reductase (NiR; At2g15620), identified in spots 45, 51 and 52, oxidizes ferredoxin for the reduction of nitrite to ammonia, which then enters the cell metabolism via the amide and amine nitrogen of amino acids (e.g. via glutamine synthetase At5g35630 in spot 55). ATP sulfurylase (ATP-S; At3g22890, spots 56 and 57), in turn, is the first enzyme of the sulfate assimilation pathway (Table 1). It catalyzes the activation of inorganic sulfate by transferring sulfate to the adenine monophosphate moiety of ATP to form the high-energy compound adenosine 5′-phosphosulfate (APS) [46]. APS is then reduced to sulfite (by APS reductase) and sulfide (by sulfite reductase), which also requires reducing equivalents of ferredoxin. Thereafter, sulfide is incorporated into cysteine, which is a precursor for methionine as well as a major component of glutathione and phytochelatins, which are needed for the induction of abiotic stress responses [47].

### Lipid metabolism

The biosynthesis of glycerolipids and fatty acids occurs in plastids (and endoplasmic reticulum). Generation of acetyl-CoA by the pyruvate dehydrogenase enzyme complex (see above) is followed by the formation of malonyl-CoA, catalyzed by acetyl-CoA carboxylase [48, 49]. In Arabidopsis, plastid acetyl-CoA carboxylase is a multienzyme complex, composed of biotin carboxylase, biotin carboxyl carrier protein, and α- and β-subunits of carboxyltransferase [50]. Indeed, three of these proteins (biotin carboxylase, At5g35360, spot 47; biotin carboxyl carrier protein, At5g16390, spot 86; carboxyl transferase subunit β, AtCg00500, spots 47 and 59) were identified in our root plastid sample (Table 1). Then, another multienzyme complex named fatty acid synthase performs cyclic condensation of two carbon units: b-ketoacyl-acyl carrier protein (ACP) synthase III (KASIII) starts fatty acid chain elongation by performing the condensation reaction of malonyl-acyl ACP and acetyl-ACP [51, 52], while KASI and KASII are the condensing enzymes for the elongation of the carbon chain from C4 to C18 [53]. KASI (At5g46290) was detected in spots 59 and 85. After the condensing reaction, the 3-ketoacyl-ACP is reduced by 3-ketoacyl-ACP reductase (also known as 3-oxoacyl-ACP reductase, At1g24360, detected in spot 97), dehydrated, and finally the enoyl-ACP reductase (At2g05990, detected in spots 82 and 83) completes the formation of saturated fatty acids [54]. The presence of these enzymes emphasizes the importance of root plastids for the fatty acid biosynthesis in Arabidopsis.

### Root mitochondria: components of citric acid cycle and respiratory electron transfer

**Fig. 4:**
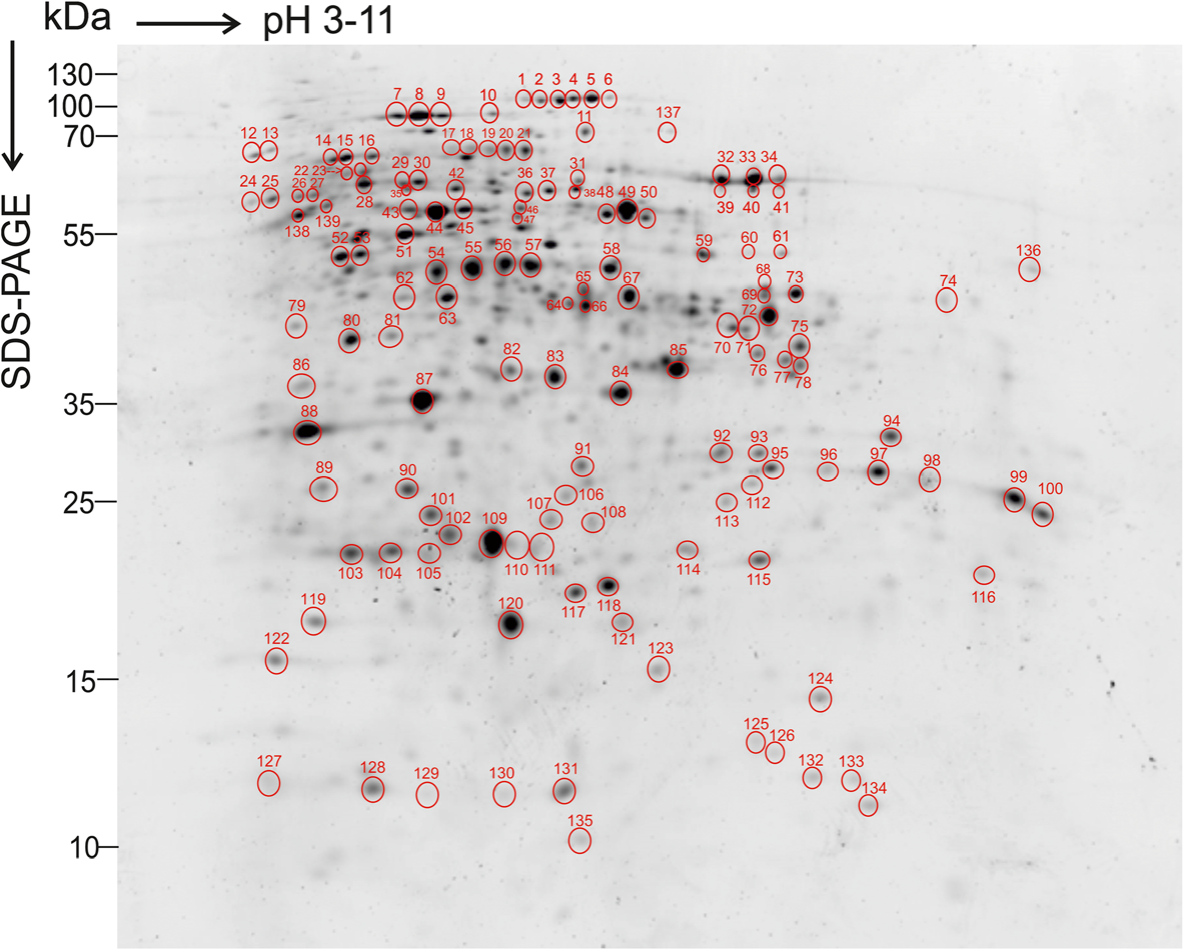
SYPRO Ruby stained 2D-gel of root protein sample enriched in plastids and mitochondria. 150 μg of proteins were separated by isoelectric focusing (pH 3–11) and SDS-PAGE (14%). The spots that were observed in three independent biological replicates are indicated in red circles. The representative gel of three independent biological replicates is shown

In line with the main role of mitochondria in producing ATP and reducing equivalents for biosynthetic reactions, 41% of the identified mitochondrial proteins were classified into the energetics category (Fig. 2, Table 2; Additional file 4: Table S2). The citric acid cycle oxidizes acetyl-CoA and produces NADH and FADH_2_, which are required for the mitochondrial electron transfer and synthesis of ATP. In our sample, multiple components of the citric acid cycle were detected. First, we identified various components of the pyruvate dehydrogenase complex producing acetyl-CoA, including dihydrolipoyl-lysine-residue acetyltransferase components 2 (At3g13930, spots 22–25, 28, 30, 79, 86, 115) and 3 (At1g54220, spots 30, 45, 86), as well as EI (pyruvate dehydrogenase E1 component subunit beta-1, At5g50850, spots 79–81, 86). In the first committed step of citric acid cycle, citrate synthase (CS) functions in converting acetyl-CoA to citrate [55]. Indeed, the CS4 (At2g44350) protein was identified in spots 56–58 and 65. Citrate is further converted to isocitrate by the aconitase (ACO) enzyme(s) [56]. In Arabidopsis, four genes encode the distinct isoforms ACO1–4. It is intriguing that the ACO2 (At4g26970) and ACO3 (At2g05710) isoforms, detected in the present study, were distributed in multiple spots indicating differential processing and/or other types of post-translational modifications of the ACO isoforms (Fig. 4, Table S1, 2). The same ACO isoforms were identified in a previous proteomic study on Arabidopsis mitochondrial proteome, but there only ACO3 was present in multiple spots [57]. Isocitrate is subsequently decarboxylated to 2-oxoglutarate by isocitrate dehydrogenase, represented by isocitrate dehydrogenase family protein (At5g14590 in spot 65) and regulatory subunits 1 (At4g35260, spot 72) and 2 (At2g17130, spots 62 and 63). 2-oxoglutarate, in turn, is decarboxylated to succinyl-CoA (by alpha-ketoglutarate dehydrogenase), which is then converted to succinate by the action of succinyl-CoA synthetase (or ligase) [58]. Succinyl-CoA ligase subunits alpha-1 (At5g08300) and beta (At 2 g20420) were identified in spots 75–78 and 85, and in spots 62 and 63, respectively. Next, succinate is oxidized to fumarate by succinate dehydrogenase complex, which participates in both the citric acid cycle and the respiratory electron transfer [59]. At5g66760, detected in spots 17, 18 and 20 encodes the flavoprotein subunit of succinate dehydrogenase, while At3g27380 in spot 97 encodes the iron-sulfur subunit 1. Fumarate is hydrated to malate (by fumarase, possibly At2g47510 in spots 59–61), which is oxidized to oxaloacetate via mitochondrial MDH. MDH1 (At1g53240) was detected in spots 83, 85 and 86, and MDH2 in spot 82.

The respiratory electron transfer chain is composed of complex I (NADH coenzyme Q reductase), complex II (succinate dehydrogenase), complex III (cytochrome bc1 complex) and complex IV (cytochrome c oxidase). Complex I is a multisubunit complex of at least 49 subunits [60], which accepts electrons in the form of NADH from the citric acid cycle. The 75 kDa protein At5g37510 (spots 1, 11, 13, 18, 21, 45, and 46) and gamma carbonic anhydrase (At1g47260 in spots 92 and 93) represented complex I (Table 2, S1). As indicated above, succinate dehydrogenase complex performs a dual function in citric acid cycle and electron transfer [61]. Complex III, in turn, was represented by prohibitin-3 (At5g40770, spots 93, 95–97, 99) and cytochrome c1 1 (At3g27240, spot 88), while we could not detect any complex IV subunits in our sample. Moreover, ATP-synthase subunits were present in multiple spots: ATP synthase subunit alpha (ATMG01190) in spots 30, 37, 43–50, 55, 56, 58, 63, 64, 79, 83, 87, 89, and 90, subunit beta-3 (At5g08680) in spots 17, 43–49, 51, 54–56, 62, and 63, subunit gamma (At2g33040) in spot 89, subunit O (At5g13450) in spot 115 and the probable subunit of 24 kDa (At2g21870) in spots 89 and 90. Distribution of the ATP synthase alpha and beta subunits to numerous spots might indicate that the assembly and/or function of the enzyme may be regulated by post-translational modifications.

Mitochondria, like all other organelles, are in constant interaction with other cellular compartments. Interaction occurs via transport of various substrates, intermediates and end-products between the compartments as well as via various signaling pathways. Porins, or voltage-dependent anion-selective channels (At5g15090, At5g67500, At3g01280), detected in multiple spots (95–97, 99, 100), enable the exchange of ions and small molecules, e.g. NADH and ATP across the mitochondrial outer membrane, thus linking mitochondrial reactions strictly to cellular metabolism.

## Discussion

### Amino acid biosynthesis in root plastids: building blocks and regulatory elements

The presence of enzymes involved in the biosynthesis of amino acids appears to be common feature among the non-green plastids [12, 13, 17]. In all higher plants L-lysine is synthesized from L-aspartate via a DAP- dependent pathway that is functional also in algae, bacteria and fungi containing cellulose in their cell wall [62]. Similarly, L-aspartate is used as a substrate also for the synthesis L-threonine. It has been shown already decades ago that intact, illuminated chloroplasts are able to synthesize all the aspartate derived amino acids from exogenous [^14^C] aspartate, indicating that they contain all the enzymes necessary for L-lysine and L-threonine synthesis [63, 64]. It is likely that the biosynthetic pathways are present also in non-green plastids, as in addition to Arabidopsis root plastids some of the involved enzymes have been found also in *Medicago truncatula* root plastids [17] and amyloplasts from developing wheat endosperm [12].

L-histidine is synthesized through a series of enzymatic reactions taking place in chloroplasts [65, 66]. As the pathway is extremely energy-consuming (41 ATP/1 His) [67], compartmentalization to chloroplasts suggests that photosynthesis might provide energy to the process [68]. However, the enzymes involved in histidine biosynthesis have also been detected in non-green plastids [12, 17]. Indeed, L-histidine has been implicated in regulation of biosynthesis of other amino acids, in chelation and transport of metal ions, and in plant reproduction and growth [69–71]. Moreover, L-histidine biosynthesis is related to a number of other metabolic pathways including synthesis of purines, pyrimidines and folates [68]. It is worth noting that also adenylosuccinate synthase (At3g57610) was detected in our analysis (Table 1). It catalyzes the first step in the de novo synthesis of adenosine monophosphate (AMP) [72], which as a purine nucleotide is a structural component of nucleic acids as well as a precursor for various vitamins, coenzymes and hormones [73–77]. Indeed, it has been shown that the plastid-localized de novo synthesis pathway is the major purine synthesis pathway in plants [73].

L-serine is an important amino acid that is an intermediate in several metabolic pathways, serves as a precursor in formation of other amino acids (like L-glycine, L-cysteine) and is involved in synthesis of phospholipids and purines [78, 79]. In plants, L-serine biosynthesis proceeds via three pathways. D-3-phosphoglycerate dehydrogenase (PGDH1), detected in multiple spots, has recently been suggested to be particularly important in non-photosynthetic tissues, as compared to the photorespiration dependent glycolate pathway functioning in the leaves [80–82].

As enzymes involved in biosynthesis of BCAAs are targets for commercially available and potential new herbicides, the biosynthetic pathways have been an object of extensive studies [26, 29]. Similarly, the loss of the shikimate pathway in animal lineages has made it an interesting research object as a source of valuable nutritional molecules and potential target of new antibiotics and herbicides. Therefore the fact that a number of enzymes involved in BCAA biosynthesis and shikimate pathway were identified in the root plastid enriched sample is particularly interesting. Recent studies have also shown that dihydroxyacid dehydratase (DHAD) is widely expressed in both vegetative and generative tissues and its activity is important for the accurate accumulation of all three BCAAs in roots [30]. Plants with reduced level of the DHAD enzyme were found to show short root phenotype as well as hypersensitivity to salt stress [30]. It should be noted that the branched-chain-amino-acid aminotransferase BCAT (and other enzymes involved in leucine biosynthesis) are also used for synthesis of glucosinolates through the methionine chain elongation pathway [83, 84], underlining the importance of root plastids for secondary metabolism and defense reactions of plants. Also polyamines and nitric oxide play important roles in the regulation of developmental processes and stress responses [85, 86]. Importantly, L-arginine is not only a precursor for both of these compounds [87], but it is also an essential amino acid for protein synthesis possessing a high nitrogen: carbon ratio. Therefore L-arginine often serves as a nitrogen storage, especially in underground storage organs and roots [88–90]. These properties position enzymes involved in L-arginine biosynthesis in the focus of current research aiming at resolving root originated stress responses.

### Impact of plastidial glycolytic pathway enzymes on primary metabolism, stress responses and developmental processes

Glycolysis is the major primary metabolic pathway in living organisms where hexoses are oxidized to provide energy, reducing power and precursors for biosynthetic reactions [91]. In plants, there are two glycolytic pathways operating in parallel in the cytosol and in the plastids, and they interact through selective transporters present in plastid envelope [92]. In addition to their role(s) in primary metabolism, several plastid enzyme isoforms appear to be involved in regulation of developmental processes and induction of stress responses. For instance, the plastidial isoform of glucose-6-phosphate isomerase has been postulated to be essential for starch synthesis in floral initiation [93] as well as for survival in low oxygen conditions [94], while the expression of the triose phosphate isomerase gene is regulated via developmental stimuli [95]. FBA, in turn, is known to be crucial both for sugar metabolism and for signaling [96]. In addition for the well-studied function of FBA 3 (At2g01140) in glycolysis, the enzyme has been suggested to be important in the induction of responses to biotic and abiotic stresses as well as in the growth and developmental processes [96]. Indeed, the mRNA level of FBA was shown to increase in response to salicylic, abscisic and gibberellic acid [96–98], drought, salt, as well as cold stress [97, 99, 100]. Interestingly, FBA 3 was localized in plastoglobules of chloroplasts and non-green plastids of Arabidopsis plants [96]. Recently, FBA has been reported as a target for lysine methylation [101] and cysteine glutathionylation [102], which may affect the conformation, activity or stability of the enzyme.

### Amyloplasts versus Arabidopsis root plastids

Several of the identified root plastid proteins, such as phosphoserine aminotransferase, arginosuccinate synthase, threonine synthase, malate dehydrogenase, fructose-bisphosphate aldolase and PPiase were also identified in the amyloplast fraction of potato tuber [10] and/or wheat ears or seeds [11, 12]. Nevertheless, the abundant proteins involved in starch and sucrose metabolism detected in wheat and potato tuber amyloplasts [10, 11] were not well represented among Arabidopsis root plastid proteins, which imply that the root plastids do not play a major role as a long-term carbohydrate storage in Arabidopsis. Alternatively, as the amyloplasts are fragile and difficult to isolate, the possibility that amyloplasts have damaged and escaped from our root plastid sample during the isolation cannot be excluded. It is also worth noting that in highly purified amyloplast fraction a number of mitochondrial proteins were identified (for instance mitochondrial porin) [10–12], which may indicate either a true dual location of some proteins, or co-purification of amyloplasts and mitochondria.

In addition to a number of plastid proteins (Table 1), also a range of mitochondrial proteins were detected, mostly representing citric acid cycle and electron transfer chain components (Table 2). In agreement with previous studies [57], many of these proteins were present as multiple spots, suggesting that mitochondrial proteins are targets of multiple post-translational modifications. Indeed, it has been recently shown that organellar proteins are frequently modified by e.g. phosphorylation [103, 104], Lys acetylation [105] and methylation [106, 107]. The functional consequences of these modifications, however, are still unknown. We also reliably identified some proteins, which may exist in multiple compartments or which have not been annotated as plastid or mitochondrial proteins. For instance jacalin-related lectins are carbohydrate binding proteins involved in defense signaling pathways [108]. In addition to the membrane-bound isoforms, plants contain soluble lectins in the cytosol, nuclei, chloroplasts as well as in the vacuole and in apoplast [109–112]. Whether the jacalin-related lectin detected in several spots (see Additional file 3; Table S1) represents a protein truly accumulating in the mitochondria or root plastids remains to be elucidated.

## Conclusions

In this study, we have identified 84 root plastid-targeted and 77 mitochondrium-targeted proteins and show that Arabidopsis root plastids have a broad biosynthetic capacity representing synthesis of amino acids and fatty acids as well as carbon, nitrogen and sulfur assimilation. This work underlines the importance of root plastids for secondary metabolism and root originated stress responses, and shows that Arabidopsis root plastids do not have a major role in long-term storage of carbohydrates. Currently we do not know whether the plastids present in our root plastid fraction represent one uniform plastid type, or multiple distinct types of leucoplasts, which may have specialized functional features. Moreover, the impact of cell or tissue types and developmental stage on the structural and functional properties of root plastids remains an interesting topic for future research.

## Methods

### Plant material

*Arabidopsis thaliana* ecotype Columbia-0 seeds, purchased from Nottingham Arabidopsis Stock Centre (NASC; UK), were surface sterilized and plated on the uppermost surface of square plates (½ x Murashige and Skoog with vitamins (Duchefa) in 50 mM MES buffer pH 5.7, 0,8% plant agar (Duchefa). The plants were grown on vertical plates under 16/8 h light/dark photoperiod at 120 μmol photons m^− 2^ s^− 1^ at 23 °C for four weeks.

### Root plastid isolation

#### SDS-PAGE and Western blotting

Root plastids were isolated as described in [113] with the following modifications. 500 mg roots of four week old plants were ground in pre-cooled mortar in 1.5 ml homogenization buffer (50 mM Tris-HCl, pH 7.5, 330 mM sorbitol, 1 mM EDTA, 1 mM MgCl2, 0,1% BSA, PierceTM Protease inhibitor, 1 tablet/10 ml). The homogenate was filtered through one layer of pre-soaked Miracloth and centrifuged (4000 x g, 3 min, 4 °C). Supernatant was collected as a cytosolic fraction, and the plastid enriched pellet was gently re-suspended in 50 μl homogenization buffer by using small brush and pipetted with a wide tip on 1.8 ml 10% Percoll followed by centrifugation (4000 x g 5 min, 4 °C). The plastid fraction was collected and washed twice with 500 μl homogenization buffer. The final pellet was re-suspended in 50 μl shock buffer (5 mM sucrose, 10 mM Hepes-NaOH, 5 mM MgCl_2_) and left on ice for 5 min.

Purity of the root plastid preparation was verified by immunodetection of cell compartment marker enzymes. Crude root homogenate, root plastid and cytosol proteins were solubilized (Laemmli, 1970) and proteins separated on 12% acrylamide gels. The gels were electroblotted onto a PVDF membrane (Immobilion-P, Merck Millipore) in blotting buffer (48 mM Tris-HCl, 39 mM glycine, 1.3 mM SDS, and 20% methanol) using 1 mA/cm^2^ for 1 h with Hoefer TE77X semidry blotter. Blots were blocked using 5% nonfat dry milk (BioRad) in TTBS (20 mM Tris-HCl, pH 7.5, 150 mM NaCl, and 0.05% Tween 20) and immunolabelled with protein-specific antibodies. Root-type FNR antibody (RFNR; gift from T. Hase, [45], dilution 1:1000) was used as a root plastid marker, and alternative oxidase1/2 (AOX1/2, Agrisera, AS04054, dilution 1: 750) and nitrate reductase (NR; Agrisera, AS08 310–100, dilution: 1:500) antibodies were used to assess the contamination by mitochondria and cytosol, respectively. Horseradish peroxidase conjugated anti-rabbit secondary antibody (GE Healthcare) with ECL Western Blotting Detection Reagents (GE Healthcare) was used for the detection of proteins on x-ray films (Fujifilm SuperRX). Blots were imaged using the Geliance 1000 imager and GeneSnap imaging software (PerkinElmer).

#### 2D gel electrophoresis and staining of gels

For 2D electrophoresis, root plastid samples of approximately 150 μg protein with rehydration buffer (8 M urea, 2 M thiourea, 4% CHAPS, 100 mM dithiothreitol, and 0.5% 3–11 NL IPG buffer) was freshly prepared, covered with aluminum foil, and vortexed with maximum speed for 2 h. A protein isoelectric focusing unit was used with NL (non-linear) 18 cm IPG strips (Bio-Rad) for pH ranges 3–11. IPG strips were rehydrated in 340 μl rehydration buffer overnight. Protein samples were cup loaded directly onto the strips, with the cup placed close to the anodic end of the strip. The first-dimension separation was performed using the Ettan IPGphor 3 isoelectric focusing system. The proteins were focused at 150 V for 3 h and at 300 V for 3 h, then the voltage was raised to 1000 V during 6 h and to 10,000 V during 2 h, and then kept at 10,000 V for 3 h [114]. After electrofocusing, the proteins in the strips were reduced with 130 mM dithiothreitol (DTT) and alkylated with 135 mM iodoacetamide (IAA) in equilibration buffer (375 mM Tris-HCl pH 8.8, 6 M urea, 20–30% glycerol, 2% SDS). Second-dimension electrophoresis was performed on 14% linear SDS gels.

After electrophoresis, gels were stained with SYPRO Ruby protein stain® according to the manufacturer’s instructions as follows. Gel fixation was performed in 40% methanol, 10% trichloroacetic acid for 3 h, followed with three washes in water for 10 min each. Subsequently, gels were covered with 330 ml of SYPRO Ruby protein stain and left overnight in darkness with continuous gentle agitation. After staining, gels were rinsed in 10% ethanol, 7% acetic acid for 45 min followed by washing in water. Stained proteins were visualized using 300 nm UV transilluminator and imaged with the Geliance 1000 imager using Cy3 filter (PerkinElmer). Thereafter, the same gels were silver stained to enable cutting of the protein spots. MS compatible silver staining was performed according to [115]. After rinsing in 20% ethanol and water for 15 min each, gels were sensitized by incubation in 200 ml 1.2 mM Na_2_S_2_O_3_ ·5 H_2_O for 90 s. Subsequently, gels were washed two times for 20 s with water and silver stained. Staining was performed in 12 mM AgNO_3_ for 30 min on the platform shaker followed by rinsing with water for 20 s. Afterwards, gels were developed in 217 mM K_2_CO_3_, 0,6 mM Na_2_S_2_O_3_, 0,07% formalin by shaking gently on the platform shaker for 2–5 min until the spots were clearly visible. The development was stopped by shaking the gels for 2 min in stop solution (2.5% acetic acid, 400 mM Tris-HCl).

#### MS analysis, database searches and functional classification

Selected protein spots from 2D gels were excised manually and subjected to in-gel digestion with 0.2–0.3 μg trypsin (Promega V5111) at 37 °C for 18 h. After digestion, tryptic peptides were extracted from the gel pieces with acetonitrile (ACN) and subsequently with 50% ACN / 5% HCOOH solution. Total extracts were pooled, dried in vacuum centrifuge and stored at − 20 °C. Directly prior to MS analyses, the peptides we dissolve in 10 μl 0.1% HCOOH by vortexing, incubating at 37 °C for 15 min. Five μl peptides were injected to LC-MS/MS analysis performed on a nanoflow HPLC system (Easy-nLC1000, Thermo Fisher Scientific) coupled to the Q Exactive mass spectrometer (Thermo Fisher Scientific, Bremen, Germany) equipped with a nano-electrospray ionization source. Peptides were first loaded on a trapping column (100 μm ID × 2 cm) and subsequently separated inline on an analytical column (75 μm ID × 15 cm). The packing material for the both columns was ReproSil-Pur 5 μm 200 Å C18-AQ (Dr. Maisch HPLC GmbH, Ammerbuch-Entringen, Germany). The mobile phase consisted of water with 0.1% formic acid (solvent A) or acetonitrile/water (80:20 (v/v)) with 0.1% formic acid (solvent B). A 10 min gradient from 8 to 43% B was used to elute peptides. MS data was acquired automatically by using Thermo Xcalibur 4.0 software (Thermo Fisher Scientific). An information dependent acquisition method consisted of an Orbitrap MS survey scan of mass range 300–1750 m/z followed by HCD fragmentation for 10 most intense peptide ions.

Data files were searched for protein identification using Proteome Discoverer 2.2 software (Thermo Fisher Scientific) connected to an in-house server running the Mascot 2.5.1 software (Matrix Science). Data was searched against SwissProt database (version 2017_03). The database search settings included a taxonomy filter ‘*Arabidopsis thaliana”*, trypsin as an enzyme, carbamido methyl as fixed modification and methionine oxidation as variable modification. Two missed cleavages were allowed. A significance threshold of *p* < 0.05 was used. Classification of proteins was performed using MapMan software (http://mapman.gabipd.org/mapman-download) and the results were manually verified using the annotations of UniProt database.

## Supplementary information

**Additional file 1: Figure S1.** Analysis of the root protein sample. The original Western blots presented in Fig. 1. 20 μg of crude root protein extract (C), the pellet representing root plastids (P) and supernatant representing cytosol (S) were separated by SDS-PAGE, transferred to a PVDF membrane and immunolabelled with root-type FNR (RFNR; root plastid marker), alternative oxidase (AOX1/2; mitochondrial marker) and nitrate reductase (NR; cytosolic marker) antibodies. kDa denotes for molecular weight markers. Arrows indicate predicted size of the proteins.

**Additional file 2: Figure S2.** 2D-gels of root protein sample enriched in plastids and mitochondria. 150 μg of proteins were separated by isoelectric focusing (pH 3–11) and SDS-PAGE (14%). A. SYPRO Ruby stained 2D-gel (also shown in Fig. 4 and Fig. S2 B) of biological replicate 1. Red circles represent protein spots analyzed by mass spectrometry. B. 2D-gels of three biological replicates. For biological replicate 1 and 2, Sypro Ruby staining is presented, and for biological replicated 3, silver staining is presented.

**Additional file 3: Table S1.** Proteins identified in root plastid enriched fraction by LC-MS/MS. Protein identification was performed using Proteome Discoverer 2.2 software as described in Methods.

**Additional file 4: Table S2.** Annotated root mitochondrial proteins identified by mass spectrometric analysis.

## Data Availability

The datasets supporting the conclusions of this article are included within the article and its additional files.

## Abbreviations

ACO: aconitase
ACP: 3-ketoacyl-acyl carrier protein
AHAS: Acetolactate synthase, acetohydroxyacid synthase
APS: adenosine 5′-phosphosulfate
BCAAs: Branched-chain amino acids
BCAT: branched-chain-amino-acid aminotransferase
CS: citrate synthase
DAP: diaminopimelic acid
DHAP: dihydroxyacetone phosphate
ENO1: Enolase 1
EPSP: 5-enolpyruvylshikimate-3-phosphate
FBA: Fructose-bisphosphatase aldolase
GAPDH: glyceraldehyde-3-phosphate dehydrogenase
G3P: glyceraldehyde 3-phosphate
Hk: homoserine kinase
KAS: ACP synthase
MDH: NAD-dependent malate dehydrogenase
PEP: phosphoenylpuryvate
PSAT: phosphoserine aminotransferase
RFNR: root-type ferredoxin-NADP^+^ oxidoreductase
TPI: Triose phosphate isomerase
TS: threonine synthase 1
