# Supplementary figures and images for: Gel-based proteomic map of Arabidopsis thaliana root plastids and mitochondria
Source: BMC Plant Biol. 2020 Sep 4;20:413. doi: 10.1186/s12870-020-02635-6 (PMC7650296; doi:10.1186/s12870-020-02635-6)

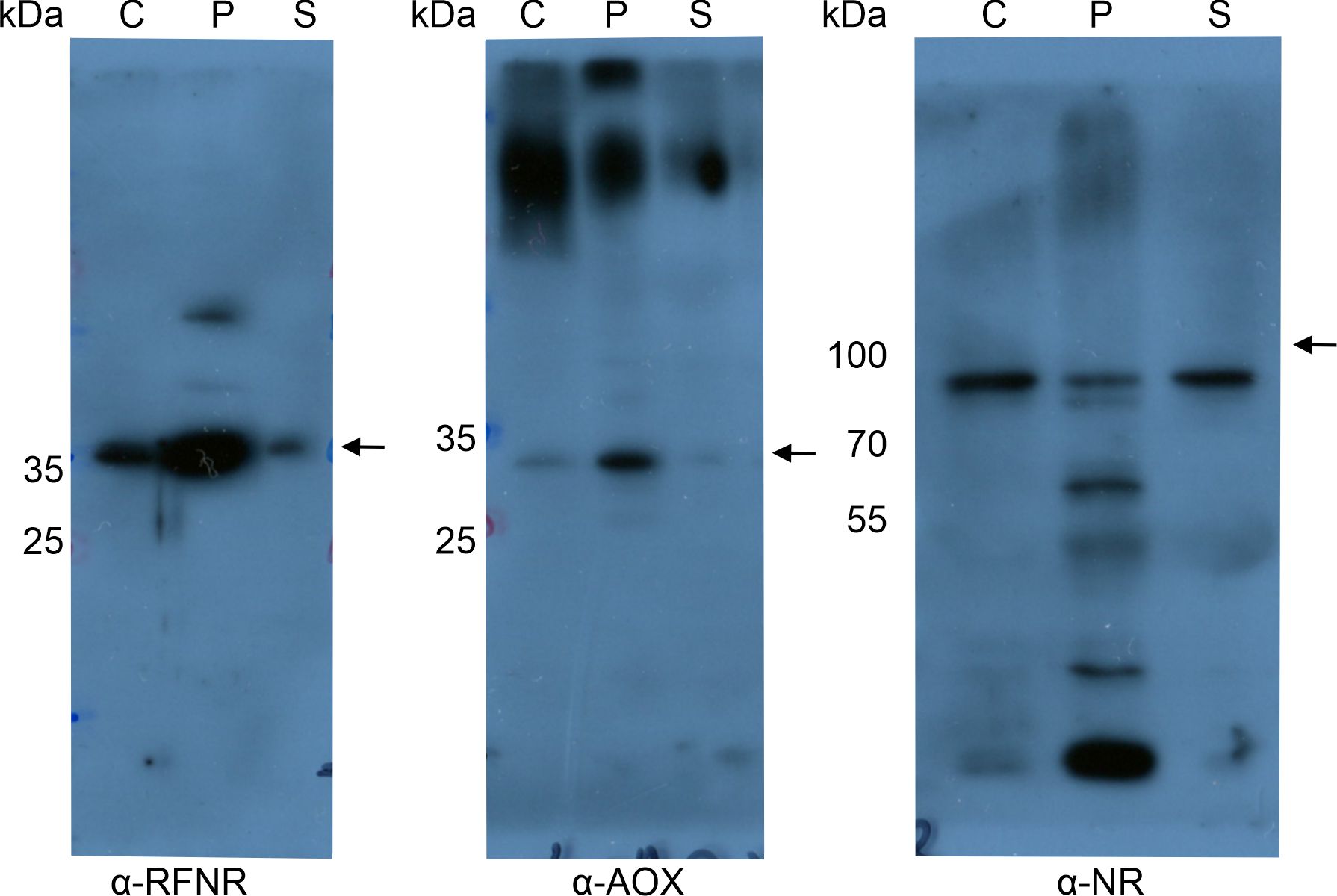

Supplement: Supplementary file 1 — Additional file 1: Figure S1. Analysis of the root protein sample. The original Western blots presented in Fig. 1. 20 μg of crude root protein extract (C), the pellet representing root plastids (P) and supernatant representing cytosol (S) were separated by SDS-PAGE, transferred to a PVDF membrane and immunolabelled with root-type FNR (RFNR; root plastid marker), alternative oxidase (AOX1/2; mitochondrial marker) and nitrate reductase (NR; cytosolic marker) antibodies. kDa denotes for molecular weight markers. Arrows indicate predicted size of the proteins. [file 12870_2020_2635_MOESM1_ESM.jpg]

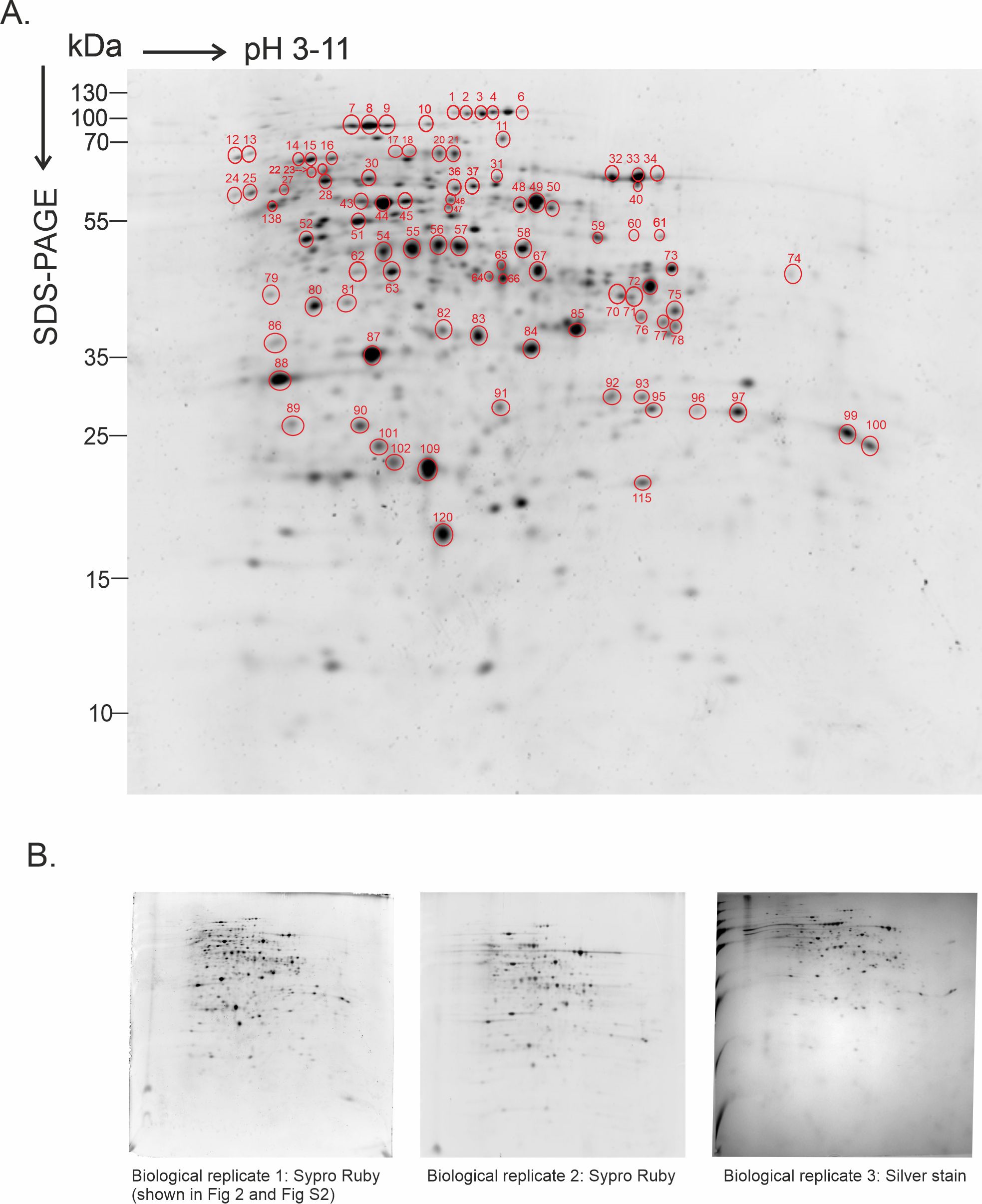

Supplement: Supplementary file 2 — Additional file 2: Figure S2. 2D-gels of root protein sample enriched in plastids and mitochondria. 150 μg of proteins were separated by isoelectric focusing (pH 3–11) and SDS-PAGE (14%). A. SYPRO Ruby stained 2D-gel (also shown in Fig. 4 and Fig. S2 B) of biological replicate 1. Red circles represent protein spots analyzed by mass spectrometry. B. 2D-gels of three biological replicates. For biological replicate 1 and 2, Sypro Ruby staining is presented, and for biological replicated 3, silver staining is presented. [file 12870_2020_2635_MOESM2_ESM.jpg]
