# Supplementary material for: Gel-based proteomic map of Arabidopsis thaliana root plastids and mitochondria
Source: BMC Plant Biol. 2020 Sep 4;20:413. doi: 10.1186/s12870-020-02635-6 (PMC7650296; doi:10.1186/s12870-020-02635-6)
Supplement: Supplementary file 4 — Additional file 4: Table S2. Annotated root mitochondrial proteins identified by mass spectrometric analysis. [file 12870_2020_2635_MOESM4_ESM.doc]

**Table 2. Annotated root mitochondrial proteins identified by mass spectrometric analysis**

| **Function** | **Spot number** | **Accession (UP),**  Gene locus (TAIR) |
| --- | --- | --- |
| | **ENERGETICS** | | | | --- | --- | --- | | *TCA cycle* | | | | Aconitate hydratase 2 | 1-4, 6, 8-11, 20, 21, 47 | Q94A28, At4g26970 | | Aconitate hydratase 3 | 1-4, 6, 10, 11, 20, 48, 54 | Q9SIB9, At2G05710 | | Citrate synthase 4 | 56- 58, 65 | P20115, At2g44350 | | Dihydrolipoyl dehydrogenase 2 | 49, 89 | Q9M5K2, At3g17240 | | Dihydrolipoyllysine-residue acetyltransferase component 2 of pyruvate dehydrogenase complex | 22-25, 28, 30, 79, 86, 115 | Q8RWN9, At3g13930 | | Dihydrolipoyllysine-residue acetyltransferase component 3 of pyruvate dehydrogenase complex | 30, 45, 86 | Q5M729, At1g54220 | | Fumarate hydratase 1 | 59- 61 | P93033, At2g47510 | | Isocitrate dehydrogenase | 65 | Q8LPJ5, At5g14590 | | Isocitrate dehydrogenase [NAD] regulatory subunit 1 | 72 | Q8LFC0, At4g35260 | | Isocitrate dehydrogenase [NAD] regulatory subunit 2 | 62, 63 | P93032, At2g17130 | | Malate dehydrogenase 1 | 83, 85, 86 | Q9ZP06, At1g53240 | | Malate dehydrogenase 2 | 82 | Q9LKA3, At3g15020 | | NAD-dependent malic enzyme 1 | 14 | Q9SIU0, At2g13560 | | NAD-dependent malic enzyme 2 | 37, 46 | Q8L7K9, At4g00570 | | Pyruvate dehydrogenase E1 component subunit beta-1 | 79- 81, 86 | Q38799, At5g50850 | | Succinate dehydrogenase | 17, 18, 20 | O82663, At5g66760 | | Succinate dehydrogenase [ubiquinone] iron-sulfur subunit 1 | 97 | Q8LBZ7, At3g27380 | | Succinate-CoA ligase [ADP-forming] subunit alpha-1 | 75- 78, 85 | P68209, At5g08300 | | Succinate-CoA ligase [ADP-forming] subunit beta | 62, 63 | O82662, At2g20420 | | Succinate-semialdehyde dehydrogenase | 44- 47 | Q9SAK4, At1g79440 | | *Electron transfer* | | | | Cytochrome c1 1 | 88 | Q9LK29, At3g27240 | | Gamma carbonic anhydrase 2 | 92, 93 | Q9C6B3, At1g47260 | | NADH dehydrogenase | 1, 11, 13, 18, 21, 45, 46 | Q9FGI6, At5g37510 | | *ATP synthesis* | | | | ATP synthase subunit alpha | 30, 37, 43- 50, 55, 56, 58, 63, 64, 79, 83, 87, 89, 90 | P92549, ATMG01190 | | ATP synthase subunit beta-3 | 17, 43- 49, 51, 54- 56, 62, 63 | Q9C5A9, AT5G08680 | | ATP synthase subunit gamma | 89 | Q96250, At2g33040 | | ATP synthase subunit O | 115 | Q96251, At5g13450 | | Probable ATP synthase 24 kDa subunit | 89, 90 | Q9SJ12, At2g21870 | | *Fermentation* | | | | Aldehyde dehydrogenase family 2 member B4 | 46- 50, 54, 57 | Q9SU63, At3g48000 | | Aldehyde dehydrogenase family 2 member B7 | 48- 50, 56, 57 | Q8S528, At1g23800 | | *Glycolysis* | | | | Plastidial pyruvate kinase 2 | 47 | Q9FLW9, At5g52920 | | **PROTEIN RELATED** | | | | Peroxisomal and mitochondrial division factor 2 | 79 | Q9SHJ6, At1g06530 | | Prohibitin-2 | 100 | Q9ZNT7, At1g03860 | | Prohibitin-3 | 93, 95- 97, 99 | O04331, At5g40770 | | *Assembly, folding* |  |  | | Chaperonin CPN60 | 22-25, 28, 30, 58, 99, 138 | P29197, At3g23990 | | Chaperonin CPN60-like 1 | 22 - 25, 28, 30, 138 | Q8L7B5, At2g33210 | | Chaperonin CPN60-like 2 | 25, 30, 43, 138 | Q93ZM7, At3g13860 | | *Degradation* |  |  | | CLP protease regulatory subunit CLPX1 | 51 | Q9FK07, At5g53350 | | Intermediate cleaving peptidase 55 | 47 | F4HZG9, At1g09300 | | *Synthesis* |  |  | | Elongation factor Ts | 81 | Q5XF75, At4g11120 | | Elongation factor Tu | 63, 64, 66 | Q9ZT91, At4g02930 | | *Targeting* |  |  | | Mitochondrial import receptor subunit TOM40-1 | 85 | Q9LHE5, At3g20000 | | Probable mitochondrial-processing peptidase subunit alpha-1 | 51, 52, 62, 79, 81 | Q9ZU25, At1g51980 | | Probable mitochondrial-processing peptidase subunit beta | 25, 30, 31, 36, 37, 45, 47, 51, 54, 79, 109 | Q42290, At3g02090 | | **AMINO ACID METABOLISM** |  |  | | *Catabolism* |  |  | | Alanine aminotransferase 1 | 43, 52 | F4I7I0, At1g17290 | | Aminomethyltransferase | 82 | O65396, At1g11860 | | Delta-1-pyrroline-5-carboxylate dehydrogenase 12A1 | 31 | Q8VZC3, At5g62530 | | Gamma-aminobutyrate transaminase POP2 | 59, 83, 85 | Q94CE5, At3g22200 | | Isovaleryl-CoA dehydrogenase | 64, 66 | Q9SWG0, At3g45300 | | Methylcrotonoyl-CoA carboxylase subunit alpha | 11, 31 | Q42523, At1g03090 | | **Ornithine aminotransferase** | 58 | Q9FNK4, At5g46180 | | *Synthesis* |  |  | | Acetylornithine aminotransferase | 62, 63 | Q9M8M7, At1g80600 | | Bifunctional L-3-cyanoalanine synthase/cysteine synthase C1 | 75- 77 | Q9S757, At3g61440 | | Cysteine synthase | 18, 81 | Q43725, At3g59760 | | Glutamine synthetase | 55 | Q43127, At5g35630 | | **STRESS RELATED** |  |  | | *Abiotic stress response* |  |  | | Chaperone protein ClpB4 | 1 | Q8VYJ7, At2g25140 | | Heat shock 70 kDa protein 10 | 14- 18, 20, 22- 25, 30 | Q9LDZ0, At5g09590 | | Heat shock 70 kDa protein 9 | 14- 18, 20, 22- 25, 30, 79 | Q8GUM2, At4g37910 | | Methylmalonate-semialdehyde dehydrogenase | 43- 47, 55 | Q0WM29, At2g14170 | | *Redox Regulation* |  |  | | Thiosulfate/3-mercaptopyruvate sulfurtransferase 1 | 86 | O64530, At1g79230 | | **CARBOHYDRATE METABOLISM** |  |  | | *Photorespiration* |  |  | | Glycine dehydrogenase 2 | 1, 2, 17, 18, 20 | O80988, At2g26080 | | Formyltetrahydrofolate deformylase 1 | 92 | Q93YQ3, At5g47435 | | **SECONDARY METABOLISM** |  |  | | Betaine aldehyde dehydrogenase 2 | 30 | Q9STS1, At3g48170 | | **MITOCHONDRION ORGANIZATION** |  |  | | Serine-tRNA ligase | 46 | Q8RWT8, At1g11870 | | **TETRAPYRROLE SYNTHESIS** |  |  | | Glutamyl-tRNA(Gln) amidotransferase subunit A | 46, 47 | Q9LI77, At3g25660 | | **LIPID METABOLISM** |  |  | | 3-hydroxyisobutyryl-CoA hydrolase-like protein 3 | 62 | Q9T0K7, At4g13360 | | Lipoamide acyltransferase component of branched-chain alpha-keto acid dehydrogenase complex | 52 | Q9M7Z1, At3g06850 | | **N METABOLISM** |  |  | | Aspartate aminotransferase | 71, 72 | P46643, At2g30970 | | Glutamate dehydrogenase 1 | 67 | Q43314, At5g18170 | | Glutamate dehydrogenase 2 | 56, 64- 67 | Q38946, At5g07440 | | **IRON SULFUR CLUSTER BIOSYNTHESIS** |  |  | | Putative transferase | 54 | Q8L733, At4g12130 | | SufE-like protein 1 | 79 | Q84W65, At4g26500 | | **C1 METABOLISM** |  |  | | Glycine dehydrogenase | 1, 2, 17, 18, 20 | O80988, At2g26080 | | **TRANSPORT** |  |  | | Mitochondrial outer membrane protein porin 2 | 97, 99, 100 | Q9FJX3, At5g67500 | | Mitochondrial outer membrane protein porin 3 | 95- 97, 99 | Q9SMX3, AT5G15090 | | Mitochondrial outer membrane protein porin1 | 97, 99 | Q9SRH5, At3g01280 | | | |
